# Is Geo-Environmental Exposure a Risk Factor for Multiple Sclerosis? A Population-Based Cross-Sectional Study in South-Western Sardinia

**DOI:** 10.1371/journal.pone.0163313

**Published:** 2016-09-26

**Authors:** Maria Cristina Monti, Davide Guido, Cristina Montomoli, Claudia Sardu, Alessandro Sanna, Salvatore Pretti, Lorena Lorefice, Maria Giovanna Marrosu, Paolo Valera, Eleonora Cocco

**Affiliations:** 1 Department of Public Health, Experimental and Forensic Medicine, Unit of Biostatistics and Clinical Epidemiology, University of Pavia, Pavia, Italy; 2 Department of Public Health, Clinical and Medical Science, University of Cagliari, Cagliari, Italy; 3 Department of Civil-Environmental Engineering and Architecture, University of Cagliari, Cagliari, Italy; 4 Department of Medical Science, University of Cagliari, Cagliari, Italy; University of Oxford, UNITED KINGDOM

## Abstract

**Background:**

South-Western Sardinia (SWS) is a high risk area for Multiple Sclerosis (MS) with high prevalence and spatial clustering; its population is genetically representative of Sardinians and presents a peculiar environment. We evaluated the MS environmental risk of specific heavy metals (HM) and geographical factors such as solar UV exposure and urbanization by undertaking a population-based cross-sectional study in SWS.

**Methods:**

Geochemical data on HM, UV exposure, urbanization and epidemiological MS data were available for all SWS municipalities. Principal Component Analysis (PCA) was applied to the geochemical data to reduce multicollinearity and confounding criticalities. Generalized Linear Mixed Models (GLMM) were applied to evaluate the causal effects of the potential risk factors, and a model selection was performed using Akaike Information Criterion.

**Results:**

The PCA revealed that copper (Cu) does not cluster, while two component scores were extracted: 'basic rocks', including cobalt, chromium and nickel, and 'ore deposits', including lead and zinc. The selected multivariable GLMM highlighted Cu and sex as MS risk factors, adjusting for age and 'ore deposits'. When the Cu concentration increases by 50 ppm, the MS odds are 2.827 (95% CI: 1.645; 5.07) times higher; females have a MS odds 2.04 times (95% CI: 1.59; 2.60) higher than males.

**Conclusions:**

The high frequency of MS in industrialized countries, where pollution by HM and CO poisoning is widespread, suggests a relationship between environmental exposure to metals and MS. Hence, we suggested a role of Cu homeostasis in MS. This is a preliminary study aimed at generating hypotheses that will need to be confirmed further.

## Introduction

The etiology of multiple sclerosis (MS) is still unknown, but it is commonly believed that genetic susceptibility combined with exposure to environmental factors are required for its development. [1] MS has an increasing incidence in populations residing at higher latitudes [2,3], and a rise in its incidence has been seen almost worldwide in the last decades, [4] pointing to the importance of changes in the environment (e.g., transition from rural to urban living, lifestyle changes) [5] interacting with a permissive genetic background.

Many genetic factors have been discovered in recent years [6], while environmental factors have been somewhat poorly identified, with the slight exception of Epstein Barr Virus (EBV), smoke, Ultraviolet (UV) exposure and vitamin D. [7–9]

Pollution and exposures to heavy metals (HM) are suspected of being involved in the pathogenesis and/or progression of various neurological diseases [10–12], including MS. [13–28]

Among such HM, aluminium (Al), barium (Ba), calcium (Ca), cadmium (Cd), copper (Cu), iron (Fe), lead (Pb), mercury (Hg), magnesium (Mg) and zinc (Zn) have been studied in MS, in different biological materials, by some case-control studies, [16–24] with not univocal results. Moreover, other suggestions have come through cluster and spatial analysis, which show a relation between trace metals and MS in different countries. [25–28]

Sardinia represents an exception to the MS latitudinal rule, by presenting a very high prevalence in spite of its geographical location. [3] Moreover, its population seems to be the ideal one to study MS etiology considering its high homogeneous genetic structures [29] enriched by MS genes [30].

In particular, it was recently shown that Southwest Sardinia (SWS) is a high-risk area for MS with a prevalence of 210.4/100,000; [31] furthermore, the analysis of geographic clustering showed an unexpectedly high MS prevalence in the upper part of the region, particularly in the municipality of Domusnovas. [31] Considering that no reasons for genetic differences of this area compared to other areas in Sardinia are present, the role of the environment could be hypothesized, considering also that the SWS economy was based in the past on the exploitation of different metals, [32] which may increase the level of trace metals released into the environment.

The SWS area is particularly rich in mineral deposits. These occurrences, arranged from the oldest to the most recent, include: i) Pb-Zn-Ba mineralisations related to Cambrian carbonatic rocks, (a large number of ore-bodies in the so-called “metalliferous ring”); ii) mineralisations with barite, iron oxides, Pb and Zn sulphides and oxides; iii) lode mineralisations with Pb, Zn, Ag and F; iv) skarns with mixed sulphides (Cu etc) and Fe; v) remobilised post-Hercynian mineralisations with Ba, Pb, Zn, Ag in Cambrian limestones.

Moreover, because of the past exploitation of the ore deposits, a lot of mining landfills have been created, which today are prone to the actions of supergene agents (e.g., the wind). Therefore, the ore-deposit-related elements are diffused around the nearer areas due to the above actions.

Given this background, we evaluated the MS environmental risk of specific HM such as Co, Cr, Ni, Cu, Pb, and Zn, and of geographical factors such as solar UV exposure and urbanization by undertaking a population-based cross-sectional study in SWS.

## Materials and Methods

### Epidemiological and environmental data

This population-based cross-sectional study was conducted in SWS (the population and area are detailed in S1 File). MS case identification methods are described elsewhere. [31]. The representativeness of the sample was guaranteed by the population-based study of Sardu et al. (2012) [33], performed on the comparable population and, following the STROBE guidelines for observational studies [34], we did not need to determine the sample size.

All MS patients gave their written informed consent to the study. The study procedures were in accordance with the Declaration of Helsinki and the study protocol was approved by the Ethical Committee of the province of Cagliari.

**Fig 1:**
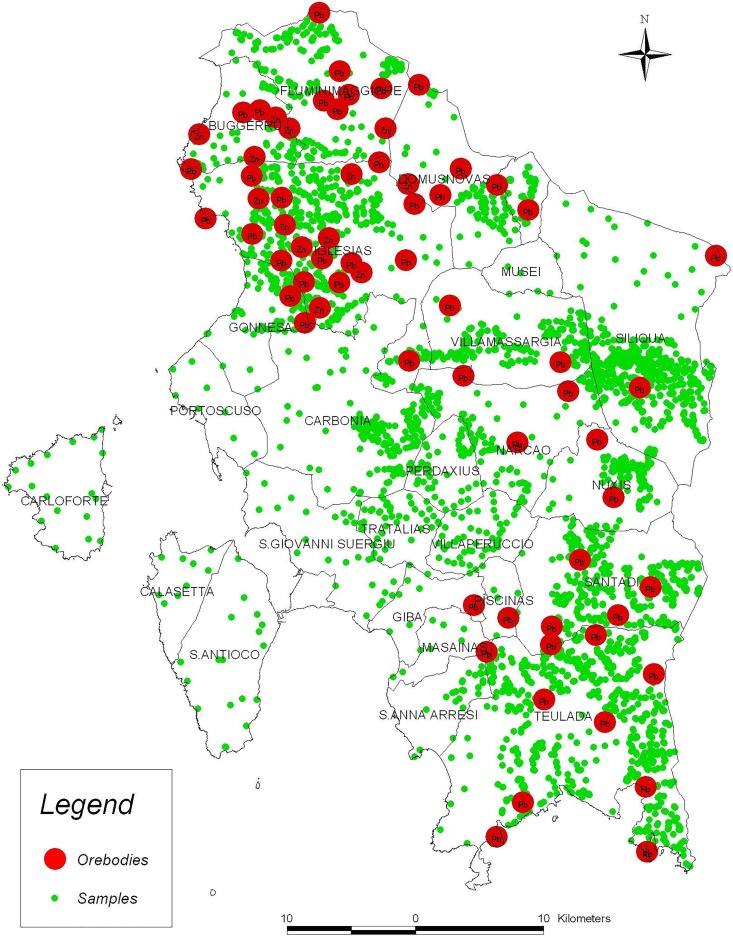
The 25 municipalities forming the study area, the sampling sites and the ore bodies.

The study considered potential environmental risk factors, classified by their geochemical and geographical nature and defined in the 25 SWS municipalities (Fig 1). We collected six HM (Co, Cr, Cu, Ni, Pb, Zn), which were revealed by geochemical samplings (Fig 1), and also a proxy of UV exposure (% of the municipal areas exposed to the south) and urbanization (% of urban area included in the municipal area), revealed by Geographic Information System processing (data collection is detailed in S2 File).

The epidemiological and environmental data matrix was created with a disaggregated structure, where the statistical units were the inhabitants of the study area. For each subject sex, age (classified in 21 five-year age classes), municipality of residence, the relative concentrations (in parts per million), UV exposure and % of urbanization were collected.

### Statistical analysis

To evaluate the marginal fixed effects of each potential risk factor (predictors), detached Generalized Linear Mixed Models (GLMMs with canonical link logit) [35] were fitted on an MS presence-vs-absence response variable. A hierarchical random effect on the municipality of residence was also included in the models to improve the fit. The model parameters were tested with the z-test. When the variability among the municipalities (random effect) was equal to zero or very small, the GLMM was reduced to a multiple logistic regression model. In addition, in order to detect multicollinearity and potential measured confounders, the pairwise associations between potential risk factors were also evaluated through Pearson correlation coefficients (r) and GLMMs as appropriate. Since HM concentrations were highly correlated, in order to reduce multicollinearity and confounding criticalities we applied a principal component analysis (PCA) [36] by synthetizing those (standardized) predictors, following Zuur et al. [37] and Suryanarayana et al. [38]. Moreover, we applied an orthogonal rotation (varimax) to the component loading matrix to obtain a simpler structure and improve interpretability. The derived “HM patterns” were quantitatively labeled according to those HM that correlated (component loadings) and loaded (component coefficients) mainly on the respective principal components, that are non-correlated between them.

Finally, we performed a multivariable GLMM (with canonical link logit) to evaluate the conditional fixed effects of the potential risk factors on MS, together with the HM principal component scores returned by the PCA. We estimated conditional fixed effects because they are the expected values, which depend on the level of the other predictors included in the model. Furthermore, we carried out an Akaike Information Criterion (AIC) model selection procedure, using a forward strategy by adding predictors to the null model. The selected GLMM had the lowest AIC. At this stage, the fixed effects of the predictors on the outcome variable are conditional; that is, we obtain the expected outcome variation (in OR terms) per unit increase of predictor, keeping fixed the others in the built-in model.

The exponential transformation of the GLMM parameters associated with the potential risk factors allowed more easily interpretable ORs to be obtained, on which P-values, 95% CIs and the measures of effect size (ES) [39] were calculated. ESs were computed to provide an estimate of the magnitude of the effects (on MS), resistant to sample size influence, whose cut-off conventions are: small if it is close to 0.2, medium if close to 0.5, and large if close to 0.8 [40].

In order to account for the very large number of the control subjects and to reduce the likelihood of identifying a statistically significant association by chance, the P-values < 0.01 were considered statistically significant, with P between 0.01 and 0.05 considered indicative of a suspect statistical significance.

Calculations were carried out using the statistical software R, version 3.2.1 [41], and its packages *lme4*, [42] *Psych* [43] and *car*. [44]

## Results

MS cases as well as HM, urbanization and UV exposure data presented an unbalanced distribution among SWS municipalities, [31] generating MS geo-environmental risk hypotheses (MS prevalence and HM concentration patterns were detailed by municipality in S1 and S3 Files).

We first tested the marginal fixed effects of the potential risk factors, i.e., Co, Cr, Cu, Ni, Pb, Zn, sun UV exposure and urbanization, sex and age on MS, in terms of crude ORs, adjusted only for the municipal random effect (Table 1). A unit increase in Cu has a significant effect on MS (OR = 1.016, 95% CI: 1.005;1.028, P = 0.006, ES = 0.009), and when Cu concentration increases by 50 ppm (corresponding approximately to the Cu range across municipalities: 10.24–64.12 ppm), the expected MS odds are 2.2 (= 1.016^50^) (95% CI: 1.28; 3.98, P = 0.006, ES = 0.436) times higher. As foreseen, females have an expected MS odds 2.03 times higher than males (95% CI: 1.6; 2.6, P<0.001, ES = 0.391). In addition, a unit increase in Cr (OR = 1.007, 95%CI: 1.000; 1.013, P = 0.038, ES = 0.004) and Ni (OR = 1.018, 95%CI: 1.001; 1.035, P = 0.040, ES = 0.010) have suspected effects on MS.

**Table 1:** Preliminary results: marginal fixed effects on MS and pairwise associations among predictors.

	Sex(1 = female vs 0 = male)	Age	Co	Cr	Ni	Cu	Pb	Zn	% municipal south exposure	% of municipal urbanization
^**1**^**Multiple Sclerosis (1 = yes vs 0 = no)**	**OR = 2.03, P<0.001 95%CI = [1.6; 2.6] ES = 0.391**	OR~1, P = 0.998 95%CI = [0.974; 1.026] ES~0	OR = 1.021, P = 0.121 95%CI = [0.995; 1.048] ES = 0.011	OR = 1.007, P = 0.038 95%CI = [1.000; 1.013] ES = 0.004	OR = 1.018, P = 0.04 95%CI = [1.001; 1.035] ES = 0.010	**OR = 1.016, P = 0.006 95%CI = [1.005; 1.028] ES = 0.009**	°OR = 0.992, P = 0.419 95%CI = [0.972; 1.012] ES = -0.004	°OR = 0.993, P = 0.300 95%CI = [0.980; 1.006] ES = -0.004	OR = 1.002, P = 0.695 95%CI = [0.990; 1.015] ES = 0.001	OR = 0.933, P = 0.431 95%CI = [0.785; 1.109] ES = -0.038
**Associations among predictors**										
^**2**^**Sex (1 = female vs 0 = male)**	-	**OR = 1.03, P<0.00195%CI = [1.02 ; 1.03]**	OR~1, P = 0.883 95%CI = [0.998; 1.002]	OR~1, P = 0.900 95%CI = [0.999; 1.001]	OR~1, P = 0.816 95%CI = [0.998; 1.002]	OR = 1.001, P = 0.278 95%CI = [0.999; 1.002]	°OR~1, P = 0.939 95%CI = [0.999; 1.002]	°OR~ 1, P = 0.874 95%CI = [0.999; 1.001]	OR~1, P = 0.426 95%CI = [0.999; 1.001]	OR = 1.01, P = 0.106 95%CI = [1;1.03]
^**2**^**Age**	-	-	Beta = -0.002, P = 0.813 95%CI = [-0.018; 0.014]	Beta = -0.002, P = 0.303 95%CI = [-0.007; 0.002]	Beta = -0.004, P = 0.471 95%CI = [-0.015; 0.007]	Beta = -0.006, P = 0.163 95%CI = [-0.015; 0.003]	Beta~0, P = 0.982 95%CI~[-0.001; 0.001]	Beta~0, P = 0.748 95%CI~[-0.001; 0.001]	Beta = -0.007, P = 0.0193 95%CI = [-0.013; -0.001]	Beta = -0.004, P = 0.947 95%CI = [-0.117; 0.110]
**Co**	-	-	-	**r = 0.67, P<0.001 95%CI = [0.66; 0.68]**	**r = 0.73, P<0.001 95%CI = [0.72; 0.74]**	**r = 0.43, P<0.001 95%CI = [0.42; 0.44]**	**r = -0.06, P<0.001 95%CI = [-0.07; -0.05]**	**r = -0.06, P<0.001, 95%CI = [-0.07; -0.05]**	**r = 0.13, P<0.001 95%CI = [0.12; 0.14]**	**r = -0.49, P<0.00 95%CI = [-0.50; -0.48]**
**Cr**	-	-	-	-	**r = 0.93, P<0.001 95%CI = [0.92; 0.94]**	**r = 0.46, P<0.001 95%CI = [0.45; 0.47]**	**r = 0.02, P<0.001 95%CI = [0.01; 0.03]**	r = 0.00, P = 0.079 95%CI = [-0.01; 0.01]	**r = 0.20, P<0.001 95%CI = [0.19; 0.21]**	**r = -0.24, P<0.001 95%CI = [-0.25; -0.23]**
**Ni**	-	-	-	-	-	**r = 0.63, P<0.001 95%CI = [0.62; 0.64]**	**r = 0.10, P<0.001 95%CI = [0.09; 0.11]**	**r = 0.09, P<0.001 95%CI = [0.08; 0.10]**	**r = 0.16, P<0.001 95%CI = [0.15; 0.17]**	**r = -0.38, P<0.001 95%CI = [-0.39; -0.37]**
**Cu**	-	-	-	-	**-**	-	**r = 0.44, P<0.001 95%CI = [0.43; 0.45]**	**r = 0.45, P<0.001 95%CI = [0.44; 0.46]**	**r = 0.26, P<0.001 95%CI = [0.25; 0.27]**	**r = -0.38, P<0.00195%CI = [-0.39; -0.37]**
**Pb**	-	-	-	-	-	-	-	**r = 0.98, P<0.001 95%CI = [0.97; 0.99]**	**r = 0.03, P<0.001 95%CI = [0.02; 0.04]**	**r = -0.07, P<0.001 95%CI = [-0.08; -0.74]**
**Zn**	-	-	-	-	-	-	-	-	**r = 0.01, P<0.001 95%CI = [0.01; 0.02]**	**r = -0.10, P<0.001 95%CI = [-0.11; -0.09]**
**% municipal south exposure**	-	-	-	-	-	-	-	-	-	**r = 0.25, P<0.001 95%CI = [0.24; 0.26]**

^1^Marginal fixed effects of potential risk factors on MS (response variable) assessed by GLMMs. OR = Odds ratio, P = P-value, 95%CI = 95% Confidence Interval, ES = Effect Size.

^2^Association assessed by GLMMs (the response variables are placed in rows; the independent variables in columns). Beta = expected response variation for unit increase of the independent variable.

r = Pearson correlation coefficient. The significance tests performed on Pearson correlation coefficients returned P-values smaller than 0.001 (except for the chromium–Zinc correlation, whose P-value was equal to 0.079) because of a high sample size (n = 138.765).The significant results (P<0.01) are shown in **bold.**

° per a 100-ppm increase.

The associations among predictors, especially among HM (Table 1), showed that Pb and Zn are highly correlated (r = 0.98), as well as Ni and Cr (r = 0.93). In addition, Cu is moderately correlated (from 0.43 to 0.63) with all the HM, and Co with Cr (r = 0.67) and Ni (r = 0.73). Furthermore, Co, Cr, Ni and Cu are negatively correlated with percentage of urbanization (range: r = -0.49 to -0.24). Finally, HM do not result associated with sex and age, which instead are associated (OR = 0.974, P<0.001, ES = -0.015). Significance tests were also performed on Pearson correlation coefficients between HM, producing P-values smaller than 0.001 (except for Cr–Zn correlation, whose P-value was equal to 0.079) because of the high sample size (n = 138,765).

**Fig 2:**
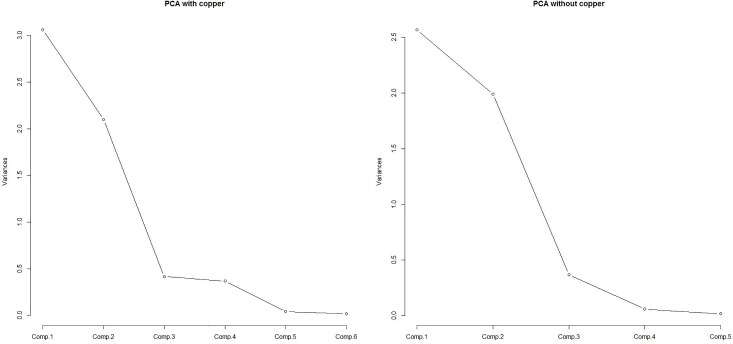
Scree plots of the principal component analysis (with and without copper).

Therefore, to perform the multivariable GLMM on MS we first applied a PCA on HM. The PCA statistics, i.e., eigenvalue>1, Horn’s parallel analysis, the very simple structure (VSS) complexity 1 = 0.90 and the scree plot (Fig 2) identified two principal components.

Table 2 shows the component loadings and coefficients (i.e., the correlation between the principal component and HM and the coefficient of the standardized HM in the principal component equation, respectively), for the two retained components after the varimax rotation. These components accounted for about 86% of the total variance in the original data. The magnitude of each loading and coefficient indicates the importance of the corresponding HM to the component. Cu is moderately correlated with both the first and second component (Table 2), and it does not univocally cluster. Since Cu is the only HM to have a significant marginal fixed effect on MS, we decided not to include it in PCA data reduction. Therefore, we ran the PCA again, with improved results: the VSS complexity 1 = 0.98, the proportion of explained variance = 0.91, and the new loadings are shown in Table 2, while the new scree plot appears in Fig 2. The first component, labeled 'basic rocks', had the greatest loadings on Ni, Cr, and Co; the second component, labeled 'ore deposits', had the greatest loadings on Pb and Zn. Consequently, we computed the two standardized component scores (the higher the scores, the greater the HM compound concentration), which were interpreted as a quantitative measure of the concentration of “basic rocks” and “ore deposits” and included in the predictor set, together with Cu, for the next GLMM analysis to evaluate the conditional fixed effects on MS.

**Table 2:** Results of principal component analysis (PCA).

	First PCA (with Cu)		Second PCA (without Cu)	
	PC1: C.l. (C.c.)	PC2: C.l. (C.c.)	PC1: C.l. (C.c.)	PC2: C.l. (C.c.)
**Label**	**“Basic Rocks”**	**“Ore Deposits”**	**“Basic Rocks”**	**“Ore Deposits”**
**Co**	**0.85 (0.302)**	-0.09 (-0.085)	**0.86 (0.336)**	-0.09 (-0.051)
**Cr**	**0.92 (0.323)**	-0.01 (-0.052)	**0.94 (0.369)**	-0.00 (-0.005)
**Ni**	**0.97 (0.333)**	0.10 (-0.006)	**0.97 (0.376)**	0.09 (0.036)
**Cu**	**0.63 (0.192)**	**0.54 (0.214)**	0.550 (—)	0.441 (—)
**Pb**	0.00 (-0.054)	**0.98 (0.445)**	0.02 (0.000)	**0.99 (0.449)**
**Zn**	-0.01 (-0.057)	**0.98 (0.448)**	0.01 (0.005)	**0.99 (0.449)**
**Proportion of Variance**	0.49	0.37	0.51	0.40
**Cumulative of Variance**	0.49	0.86	0.51	0.91

The **bold** values relate the representative heavy metals allocated to the two PCs.

C.l. = component loadings; i.e., correlation between principal component and heavy metal.

C.c. = component coefficients; i.e., coefficient of the standardized heavy metals in principal component equation.

Concerning the correlations among the HM, from a geochemical point of view, Co-Cr-Ni associations (i.e., the first principal component score) are well known. Indeed, these associations are typical of the so-called "basic or ultrabasic rocks", that is, rocks with a comparatively low Silica content. There is a nearly total correspondence between high values for Co-Cr-Ni and the occurrence of these rocks. In addition, as previously described, the area is particularly rich in Pb-Zn ore bodies, and thus the second principal component score is closely related to those ores.

Finally, the predictor set of the multivariable GLMM included the two principal component scores, Cu, sex and age, and the two municipal percentages. Sex was included because its marginal fixed effect on MS was significant, while age was included as an adjustment covariate because of its biological relevance. Table 3 shows the fitted models. The selected model (Model 3) did not encompass the predictors 'basic rocks' and the two UV exposure and urbanization percentages. At this stage, the fixed effects of the predictors on the outcome variable are conditional; in other words, we speak of the expected outcome variation (in OR terms) per unit increase of predictor, keeping fixed the other predictors in the built-in model. Therefore, we note that sex and Cu continued to be significant risk factors. Adjusting for the other predictors, females have an expected MS odds 2.04 (95%CI: 1.59; 2.60, P<0.001, ES = 0.394) times higher than males and, when Cu concentrations increase by 50 ppm, the expected MS odds are 2.827 (= 1.021^50^) (95% CI: 1.645; 5.07, P<0.001, ES = 0.574) times higher (the OR per ppm unit increase of Cu concentration is 1.021, 95% CI: 1.010; 1.033, P<0.001, ES = 0.011).

**Table 3:** Multivariable GLMMs results.

Model	AIC	Predictors							Random effect variance
		Age	Sex	Cu	'Ore Deposits'	Percentage of urbanization	'Basic Rocks'	Percentage of south exposure	
Model 1	4147.638	OR = 0.995, P = 0.730 95%CI = [0.970; 1.022] ES = -0.003	**OR = 2.03, P<0.001 95%CI = [1.59; 2.60] ES = 0.391**	-	-	-	-	-	0.114
Model 2	4143.629	OR = 0.996, P = 0.739 95%CI = [0.970; 1.022] ES = -0.002	**OR = 2.04, P<0.001 95%CI = [1.59; 2.60] ES = 0.394**	**OR = 1.016, P = 0.005 95%CI = [1.004; 1.028] ES = 0.009**	-	-	-	-	0.044
Model 3*	4140.397	OR = 0.996, P = 0.736 95%CI = [0.970; 1.022] ES = -0.002	**OR = 2.04, P<0.001 95%CI = [1.59; 2.60] ES = 0.394**	**OR = 1.021, P<0.001 95%CI = [1.010; 1.033] ES = 0.011**	OR = 0.836, P = 0.031 95%CI = [0.710; 0.984] ES = -0.099	-	-	-	0.028
Model 4	4142.063	OR = 0.996, P = 0.737 95%CI = [0.970; 1.022] ES = -0.002	**OR = 2.04, P<0.001 95%CI = [1.59; 2.61] ES = 0.394**	**OR = 1.023, P<0.001 95%CI = [1.010; 1.035] ES = 0.013**	OR = 0.828, P = 0.027 95%CI = [0.700; 0.979] ES = -0.104	OR = 1.041, P = 0.561 95%CI = [0.909; 1.192] ES = 0.022	-		0.026
Model 5	4142.234	OR = 0.995 P = 0.733 95%CI = [0.970; 1.022] ES = -0.003	**OR = 2.04, P<0.001 95%CI = [1.59; 2.60] ES = 0.394**	**OR = 1.019, P = 0.009 95%CI = [1.005; 1.034] ES = 0.010**	OR = 0.848, P = 0.060 95%CI = [0.714; 1.007] ES = -0.091	-	OR = 1.040, P = 0.680 95%CI = [0.863; 1.253] ES = 0.022	-	0.023
Model 6	4142.393	OR = 0.996 P = 0.735 95%CI = [0.970; 1.022] ES = -0.002	**OR = 2.04, P<0.001 95%CI = [1.59; 2.60] ES = 0.394**	**OR = 1.022, P<0.001 95%CI = [1.009; 1.034] ES = 0.012**	OR = 0.835, P = 0.031 95%CI = [0.709; 0.984] ES = -0.100	-	-	OR = 0.999, P = 0.947 95%CI = [0.989; 1.010] ES = -0.001	0.028

*selected model

OR = Odds ratio, P = P-value, 95%CI = 95% Confidence Interval, ES = Effect Size. The significant results (P<0.01) are shown in **bold.**

In addition, in order to check again the multicollinearity of the selected GLMM, we computed the generalized variance inflation factors (gVIF) [35] for each predictor: all the explanatory variables returned a gVIF less than 2, thereby confirming the adequacy of the model regarding its parameter estimates.

## Discussion

Taking advantage of the availability of reliable MS epidemiological, geographical and geochemical data in SWS, we performed a population-based cross-sectional study to explore geo-environmental risk hypotheses generated from the evidence of high and non-homogeneous MS prevalence in SWS.

We obtained associations of Cu levels and sex with MS distribution. In particular, we found that when Cu concentrations increase by 50 ppm, the adjusted MS odds are almost 3 times higher. Moreover, among the HM searched for in the study area, the geographical distribution of Cu levels (S3 File) turned out to be well associated with the peculiar MS prevalence in two villages of SWS. [31] Specifically, the village of Domusnovas has a high value of Cu (64.12±18.44 ppm) and a high MS prevalence (431 per 100,000), while the low MS prevalence in Carloforte is associated with low Cu (10.24±18.26 ppm). It has been recently demonstrated that the Sardinian general population presents a high MS genetic load with respect to other Italian populations. [30] No reason for the genetic differences with respect to other Sardinians exists in Domusnovas; conversely, people living in Carloforte (of Tabarkin origin) have a different genetic background. However, it could be hypothesized that environmental factors, such as Cu levels in the environment, along with the expression of genetic variants present in the two populations and based on a specific environmental trigger (as recently evidenced for Vitamin D levels), [45] may play a role in MS differences in the two villages.

Therefore, the possible association of Cu and MS has some biologically plausible basis. Cu homeostasis is fundamental in maintaining the essential function of enzymes and in avoiding the generation of toxic reactive oxygen species. In particular, Cu excess could be detrimental to the brain function and has been associated with the neurodegenerative process in humans. [46] The co-association of MS with Wilson's disease has been reported in a few cases [47]; moreover, high Cu levels in the cerebrospinal fluid (CSF) have been associated with the demyelinating process in both the central and peripheral nervous systems regarding Skogholt disease. [48] Additionally, one of the most relevant animal models of demyelination is obtained by the administration of cuprizone, [49] a Cu chelating mitochondrial toxin causing oligodendrocytes apoptosis and demyelination in the CNS. [50] Furthermore, the use of clioquinol, a Cu/Zn chelator, determined a reduction of the white matter damage in the spinal cord of an MS model in mice. [51] In addition, the environmental component as measured by Cu levels has been dosed in different biological matrices (CSF, blood, hairs) of MS patients and controls, but no definitive results have been reached [17,18, 20, 22–24], probably because of the small sample sizes.

Concerning the sex effect, our results are in line with the well-known sex bias in MS. In recent decades there has been some evidence of an increase in the sex gap in MS; [4] interestingly, in Crete a marked increase in MS incidence has been described, especially among women living in urban areas or who have moved at a young age from rural to urban areas. [5]

However, the way in which the female sex and Cu levels could influence MS risk or interact is elusive, and we cannot speculate on this due to the geo-environmental nature of this study. Moreover, it is interesting to note that the observed excess of MS risk in males from Domusnovas [28] could not be explained only by the Cu values in the territory. We are planning further studies to try to answer this question. Finally, we also included UV exposure and urbanization, but these factors did not turn out to be significantly associated with the MS distribution.

This study was performed using a population-based study design in a very well-characterized and informative area; the epidemiological data used is very reliable since it derived not only from administrative sources but also from a matching with clinical data collected by MS specialists. It should be noted that very few studies have looked for the association between MS and metals at population levels; [25–28] similarly, it has recently been suggested that the causes of MS are pervasive across all population groups, and investigating etiological factors operating at the population level could be more informative than searching for local-level causes of the disease. [52]. Finally, the ultimate fixed effects of the MS risk factors reported were conditional (by using GLMM, which also included a hierarchical random effect on the municipality of residence). This contributed to a decrease in the heterogeneity bias, a common error in observational studies that might cause contradictions.

On the other hand, we have to take into consideration some limitations of the research and the fact that the results are not conclusive but should be considered cautiously as hypothesis generating. Data should be confirmed for the whole island and also for different geographic regions. Furthermore, we have suggested there is an association at the population level but not at the individual one. This issue suggests caution in generalizing these results to a world-wide level, and a case-control study is underway comparing HM levels in biological samples from MS and healthy individuals.

In addition, it is also important to underline that we considered MS prevalence data, which gives an idea of momentary but not past environmental exposures, which instead incidence data can reveal, being closer in time to the onset moment. Moreover, the present model of analysis does not take into account other factors that potentially influence MS occurrence, such as smoking habits, EBV positivity, vitamin D levels, and specific genetic factors; however we are planning to consider these in a future analysis. For example, confirmatory statistical models, in a multivariate Structural Equation Models framework, [53] might be useful to model gene-environment interactions by evaluating the causal effects of (selected) environmental factors on MS, mediated and/or moderated by (selected) genetic markers.

Finally, since this is an observational study, it is worth pointing out any issues concerning potential unmeasured confounders. As is well known in the mineral prospecting field, in the natural environment some elements are highly intercorrelated. Moreover, some elements associated within the same group are geochemically more mobile than others, and this higher mobility is used in the mineral prospecting field to identify, sometimes kilometers upstream, mineral deposits for the less-mobile elements. This also means that analytical information about a given element is available in a territory whose lithological and mining characteristics are known. Therefore, the diffusion of other associated elements can be hypothesized with a good degree of approximation. In this specific case, data of a limited number of elements was processed, and Cu was found to have a significant effect on MS. However, we must consider the other elements associated with Cu in the local geological context, even if they were unmeasured. This fact clearly brings out that it will take more studies before this fact can be better explained.

## Supporting Information

S1 File SWS MS data and population. (DOC)

S2 File Study area and geo-data. (DOC)

S3 File Heavy metals and geographical factors. (DOC)
