# Supplementary material for: Is Geo-Environmental Exposure a Risk Factor for Multiple Sclerosis? A Population-Based Cross-Sectional Study in South-Western Sardinia
Source: PLoS One. 2016 Sep 26;11(9):e0163313. doi: 10.1371/journal.pone.0163313 (PMC5036813; doi:10.1371/journal.pone.0163313)
Supplement: S1 File — (DOC) [file pone.0163313.s001.doc]

***S2 File. SWS MS data and population***

We reported SWS data by municipality, age and gender (Table 2S). The MS prevalence was 210.4/100,000, updated to 31 December 2007 (Cocco et al., 2011) and defined by counting the number of patients diagnosed on that day. Females showed an overall MS prevalence that was higher than males (280.3 vs 138); the highest municipality prevalences were observed in Piscinas (474.5) and Domusnovas (431.5). The female strata in Piscinas and Villaperuccio and the male stratum in Domusnovas reveal the highest prevalences, equal to 696, 563 and 465, respectively. Furthermore, the highest prevalences were in the 25 to 54 year old age groups (range: 321 to 455). In particular, the highest values of prevalence (455.25) was in the 45-49 year old group, corresponding to 278.34 for the male stratum and 625.67 for the female one.

**Table 2S. MS prevalence and absolute frequencies of reference population by municipality, classes of age and gender**

| **Municipality** | **MS prevalence x 100,000** | | | **Reference population by age class and gender** | | | | | | | | | | | | | | | | | |
| --- | --- | --- | --- | --- | --- | --- | --- | --- | --- | --- | --- | --- | --- | --- | --- | --- | --- | --- | --- | --- | --- |
|  |  |  |  | **0-4** | | **5-9** | | **10-14** | | **15-19** | | **20-24** | | **25-29** | | **30-34** | | **35-39** | | **40-44** | |
|  | **Overall** | **M** | **F** | **M** | **F** | **M** | **F** | **M** | **F** | **M** | **F** | **M** | **F** | **M** | **F** | **M** | **F** | **M** | **F** | **M** | **F** |
| **BUGGERRU** | 0 | 0 | 0 | 20 | 11 | 19 | 14 | 21 | 23 | 32 | 27 | 28 | 36 | 36 | 33 | 48 | 42 | 31 | 31 | 37 | 40 |
| **CALASETTA** | 104.38 | 71.37 | 135.77 | 40 | 52 | 40 | 49 | 55 | 45 | 87 | 59 | 61 | 70 | 99 | 89 | 129 | 105 | 106 | 95 | 99 | 92 |
| **CARBONIA** | 215.76 | 117.14 | 307.44 | 463 | 488 | 562 | 518 | 652 | 550 | 810 | 806 | 909 | 850 | 1069 | 978 | 1203 | 1143 | 998 | 1052 | 1027 | 1118 |
| **CARLOFORTE** | 46.37 | 31.27 | 61.14 | 133 | 114 | 134 | 108 | 110 | 110 | 130 | 134 | 148 | 175 | 211 | 177 | 260 | 220 | 259 | 220 | 232 | 236 |
| **DOMUSNOVAS** | 431.5 | 465.12 | 398.28 | 126 | 95 | 138 | 121 | 156 | 148 | 205 | 199 | 227 | 202 | 228 | 231 | 255 | 244 | 222 | 234 | 252 | 272 |
| **FLUMINIMAGGIORE** | 132.01 | 134.68 | 129.45 | 42 | 50 | 67 | 68 | 73 | 60 | 83 | 87 | 81 | 86 | 82 | 94 | 88 | 85 | 98 | 115 | 133 | 119 |
| **GIBA** | 329.87 | 287.63 | 370.72 | 32 | 35 | 33 | 39 | 43 | 31 | 61 | 49 | 62 | 65 | 71 | 67 | 69 | 71 | 75 | 81 | 95 | 93 |
| **GONNESA** | 173.18 | 154.02 | 192.31 | 107 | 89 | 95 | 83 | 106 | 95 | 175 | 139 | 163 | 174 | 183 | 173 | 208 | 187 | 191 | 190 | 197 | 193 |
| **IGLESIAS** | 256.48 | 158.06 | 347.32 | 480 | 432 | 503 | 525 | 602 | 551 | 762 | 761 | 865 | 793 | 912 | 830 | 1056 | 1003 | 1009 | 1084 | 1128 | 1141 |
| **MASAINAS** | 215.98 | 147.06 | 282.09 | 21 | 16 | 19 | 18 | 23 | 26 | 36 | 38 | 48 | 37 | 44 | 47 | 43 | 51 | 51 | 42 | 50 | 59 |
| **MUSEI** | 66.49 | 0 | 137.17 | 25 | 22 | 32 | 24 | 45 | 33 | 46 | 50 | 62 | 54 | 67 | 55 | 54 | 52 | 49 | 48 | 50 | 58 |
| **NARCAO** | 147.58 | 118.06 | 177.1 | 51 | 61 | 74 | 63 | 77 | 85 | 101 | 99 | 114 | 94 | 121 | 108 | 108 | 114 | 133 | 131 | 128 | 141 |
| **NUXIS** | 175.13 | 115.87 | 235.29 | 28 | 22 | 26 | 38 | 43 | 36 | 50 | 45 | 51 | 40 | 56 | 41 | 71 | 57 | 55 | 65 | 58 | 64 |
| **PERDAXIUS** | 205.48 | 136.61 | 274.72 | 14 | 18 | 32 | 25 | 28 | 42 | 39 | 37 | 45 | 45 | 56 | 40 | 43 | 50 | 77 | 55 | 56 | 63 |
| **PISCINAS** | 474.5 | 242.71 | 696.06 | 16 | 15 | 19 | 11 | 23 | 17 | 27 | 26 | 26 | 29 | 19 | 24 | 33 | 31 | 28 | 32 | 28 | 37 |
| **PORTOSCUSO** | 244.22 | 150.77 | 337.08 | 97 | 97 | 105 | 88 | 99 | 96 | 127 | 130 | 175 | 144 | 189 | 189 | 234 | 224 | 221 | 201 | 205 | 212 |
| **S. ANNA ARRESI** | 37.34 | 73.37 | 0 | 118 | 101 | 124 | 101 | 159 | 145 | 159 | 136 | 194 | 179 | 230 | 223 | 253 | 228 | 184 | 183 | 234 | 229 |
| **S. ANTIOCO** | 135.93 | 102.44 | 169.09 | 63 | 61 | 66 | 80 | 73 | 62 | 104 | 114 | 119 | 119 | 121 | 113 | 137 | 115 | 131 | 131 | 128 | 133 |
| **S. GIOVANNI SUERGIU** | 164.52 | 98.36 | 231.18 | 45 | 47 | 54 | 37 | 58 | 53 | 86 | 70 | 77 | 85 | 105 | 96 | 97 | 93 | 97 | 99 | 103 | 101 |
| **SANTADI** | 243.18 | 164.11 | 320.34 | 202 | 176 | 257 | 209 | 263 | 229 | 274 | 268 | 324 | 316 | 386 | 364 | 447 | 446 | 422 | 408 | 469 | 430 |
| **SILIQUA** | 319.57 | 197.53 | 440.53 | 73 | 74 | 77 | 84 | 88 | 89 | 124 | 113 | 141 | 140 | 140 | 126 | 151 | 146 | 152 | 151 | 170 | 133 |
| **TEULADA** | 208.27 | 51.22 | 370.57 | 66 | 55 | 52 | 44 | 63 | 68 | 83 | 89 | 101 | 79 | 118 | 103 | 156 | 114 | 142 | 140 | 164 | 135 |
| **TRATALIAS** | 178.89 | 0 | 355.87 | 20 | 18 | 22 | 23 | 26 | 21 | 24 | 30 | 45 | 40 | 41 | 42 | 41 | 28 | 33 | 48 | 49 | 41 |
| **VILLAMASSARGIA** | 216.74 | 108.28 | 325.38 | 73 | 63 | 104 | 52 | 92 | 87 | 86 | 102 | 114 | 112 | 140 | 136 | 140 | 138 | 131 | 154 | 159 | 138 |
| **VILLAPERUCCIO** | 276.24 | 0 | 562.85 | 24 | 16 | 19 | 14 | 18 | 20 | 28 | 30 | 42 | 28 | 32 | 39 | 44 | 37 | 43 | 38 | 42 | 41 |
| **Total reference population (MS cases)** | 138765 (292) | 68138 (94) | 70627 (198) | 2379 (0) | 2228 (0) | 2673 (0) | 2436 (0) | 2996 (0) | 2722 (0) | 3739 (1) | 3638 (0) | 4222 (2) | 3992 (11) | 4756 (5) | 4418 (25) | 5368 (15) | 5024 (23) | 4938 (14) | 5028 (18) | 5293 (8) | 5319 (30) |
| **Total prevalence per 100,000** | **210.43** | **137.96** | **280.35** | **0** | **0** | **0** | **0** | **0** | **0** | **26.7** | **0** | **47.3** | **275.5** | **105.1** | **565.8** | **279.4** | **457.8** | **283.5** | **357.9** | **151.1** | **564.0** |

| **Municipality** | **Reference population by age class and gender** | | | | | | | | | | | | | | | | | | | | | | | |
| --- | --- | --- | --- | --- | --- | --- | --- | --- | --- | --- | --- | --- | --- | --- | --- | --- | --- | --- | --- | --- | --- | --- | --- | --- |
|  | **45-49** | | **50-54** | | **55-59** | | **60-64** | | **65-69** | | **70-74** | | **75-79** | | **80-84** | | **85-89** | | **90-94** | | **95-99** | | **100-104** | |
|  | **M** | **F** | **M** | **F** | **M** | **F** | **M** | **F** | **M** | **F** | **M** | **F** | **M** | **F** | **M** | **F** | **M** | **F** | **M** | **F** | **M** | **F** | **M** | **F** |
| **BUGGERRU** | 51 | 49 | 41 | 44 | 44 | 46 | 26 | 27 | 32 | 34 | 18 | 34 | 32 | 36 | 25 | 33 | 7 | 8 | 2 | 5 | 0 | 1 | 0 | 0 |
| **CALASETTA** | 92 | 95 | 99 | 121 | 119 | 114 | 104 | 103 | 93 | 105 | 58 | 79 | 46 | 73 | 33 | 70 | 30 | 38 | 9 | 13 | 2 | 6 | 0 | 0 |
| **CARBONIA** | 1078 | 1208 | 1253 | 1353 | 1341 | 1352 | 975 | 967 | 719 | 829 | 497 | 664 | 437 | 679 | 302 | 574 | 154 | 324 | 50 | 131 | 14 | 23 | 0 | 6 |
| **CARLOFORTE** | 226 | 216 | 199 | 215 | 212 | 234 | 244 | 251 | 190 | 225 | 175 | 178 | 143 | 181 | 107 | 132 | 66 | 91 | 16 | 42 | 3 | 11 | 0 | 1 |
| **DOMUSNOVAS** | 286 | 267 | 241 | 228 | 239 | 224 | 197 | 183 | 137 | 167 | 104 | 140 | 113 | 160 | 69 | 78 | 18 | 49 | 11 | 17 | 1 | 4 | 0 | 1 |
| **FLUMINIMAGGIORE** | 140 | 137 | 125 | 109 | 120 | 91 | 71 | 78 | 80 | 91 | 68 | 81 | 70 | 95 | 47 | 62 | 13 | 28 | 3 | 8 | 1 | 1 | 0 | 0 |
| **GIBA** | 82 | 85 | 88 | 68 | 80 | 86 | 62 | 63 | 60 | 73 | 53 | 53 | 45 | 62 | 15 | 40 | 8 | 15 | 9 | 3 | 0 | 0 | 0 | 0 |
| **GONNESA** | 203 | 224 | 249 | 225 | 210 | 203 | 138 | 123 | 129 | 126 | 95 | 98 | 64 | 131 | 53 | 87 | 24 | 39 | 7 | 17 | 0 | 4 | 0 | 0 |
| **IGLESIAS** | 1115 | 1255 | 1079 | 1083 | 1024 | 1048 | 733 | 859 | 602 | 816 | 484 | 657 | 466 | 686 | 292 | 483 | 123 | 258 | 36 | 104 | 14 | 22 | 1 | 5 |
| **MASAINAS** | 61 | 67 | 63 | 55 | 58 | 48 | 40 | 44 | 26 | 45 | 36 | 42 | 24 | 31 | 25 | 22 | 7 | 11 | 5 | 10 | 0 | 0 | 0 | 0 |
| **MUSEI** | 73 | 64 | 65 | 60 | 62 | 59 | 51 | 31 | 26 | 45 | 22 | 26 | 27 | 20 | 12 | 17 | 5 | 9 | 2 | 2 | 0 | 0 | 0 | 0 |
| **NARCAO** | 150 | 132 | 133 | 118 | 121 | 102 | 99 | 87 | 100 | 100 | 57 | 76 | 62 | 71 | 49 | 63 | 14 | 30 | 1 | 15 | 1 | 3 | 0 | 1 |
| **NUXIS** | 71 | 64 | 66 | 67 | 72 | 54 | 44 | 46 | 42 | 54 | 47 | 49 | 39 | 44 | 24 | 28 | 14 | 20 | 4 | 10 | 1 | 6 | 1 | 0 |
| **PERDAXIUS** | 56 | 52 | 59 | 54 | 55 | 53 | 41 | 32 | 43 | 36 | 29 | 41 | 22 | 34 | 20 | 27 | 11 | 15 | 5 | 6 | 1 | 3 | 0 | 0 |
| **PISCINAS** | 31 | 33 | 27 | 28 | 30 | 31 | 24 | 23 | 22 | 27 | 18 | 20 | 17 | 22 | 16 | 16 | 6 | 4 | 2 | 5 | 0 | 0 | 0 | 0 |
| **PORTOSCUSO** | 193 | 185 | 208 | 225 | 206 | 220 | 196 | 163 | 150 | 152 | 119 | 123 | 56 | 91 | 40 | 70 | 21 | 37 | 9 | 15 | 3 | 6 | 0 | 2 |
| **S. ANNA ARRESI** | 234 | 243 | 246 | 236 | 274 | 247 | 181 | 168 | 163 | 170 | 100 | 145 | 94 | 138 | 65 | 98 | 21 | 41 | 12 | 14 | 5 | 3 | 0 | 0 |
| **S. ANTIOCO** | 152 | 151 | 142 | 142 | 140 | 115 | 100 | 107 | 106 | 125 | 78 | 92 | 71 | 95 | 51 | 65 | 28 | 35 | 14 | 14 | 4 | 4 | 0 | 0 |
| **S. GIOVANNI SUERGIU** | 100 | 112 | 115 | 110 | 107 | 98 | 97 | 64 | 65 | 81 | 56 | 52 | 45 | 47 | 31 | 37 | 18 | 21 | 6 | 10 | 1 | 2 | 0 | 0 |
| **SANTADI** | 440 | 421 | 429 | 424 | 437 | 443 | 383 | 393 | 337 | 369 | 296 | 314 | 249 | 294 | 143 | 217 | 65 | 135 | 27 | 41 | 7 | 16 | 0 | 1 |
| **SILIQUA** | 167 | 150 | 158 | 170 | 149 | 144 | 97 | 111 | 108 | 100 | 81 | 109 | 77 | 103 | 44 | 59 | 19 | 22 | 7 | 15 | 1 | 4 | 1 | 0 |
| **TEULADA** | 172 | 157 | 179 | 135 | 143 | 126 | 120 | 113 | 97 | 101 | 107 | 113 | 81 | 128 | 62 | 94 | 31 | 64 | 12 | 18 | 3 | 12 | 0 | 1 |
| **TRATALIAS** | 43 | 37 | 40 | 43 | 50 | 44 | 29 | 27 | 28 | 29 | 22 | 27 | 18 | 34 | 17 | 17 | 8 | 10 | 0 | 2 | 0 | 1 | 0 | 0 |
| **VILLAMASSARGIA** | 139 | 143 | 147 | 143 | 132 | 130 | 96 | 115 | 99 | 90 | 73 | 86 | 66 | 69 | 37 | 51 | 13 | 20 | 5 | 12 | 1 | 2 | 0 | 1 |
| **VILLAPERUCCIO** | 34 | 47 | 46 | 41 | 48 | 37 | 26 | 39 | 40 | 34 | 18 | 16 | 15 | 24 | 16 | 23 | 11 | 5 | 7 | 4 | 0 | 0 | 0 | 0 |
| **Total reference population (MS cases)** | 5389 (15) | 5594 (35) | 5497 (17) | 5497 (20) | 5473 (9) | 5349 (17) | 4174 (3) | 4217 (10) | 3494 (3) | 4024 (8) | 2711 (1) | 3315 (1) | 2379 (0) | 3348 (0) | 1595 (1) | 2463 (0) | 735 (0) | 1329 (0) | 261 (0) | 533 (0) | 63 (0) | 134 (0) | 3 (0) | 19 (0) |
| **Total prevalence per 100,000** | **278.3** | **625.6** | **309.2** | **363.8** | **164.4** | **317.8** | **71.8** | **237.1** | **85.8** | **198.8** | **36.8** | **30.1** | **0** | **0** | **62.6** | **0** | **0** | **0** | **0** | **0** | **0** | **0** | **0** | **0** |

M= males, F= females, MS=Multiple Sclerosis

Because some municipalities returned a number of MS cases < 3, cases by single municipality are not shown in the respect of privacy and subject identification italian legislation (Codice di deontologia e buona condotta per il trattamento dei dati personali per scopi di ricerca statistici e scientifici - Provvedimento del Garante per la Privacy, Gazzetta Ufficiale n. 190 del 14 agosto 2004).
