# Supplementary material for: Is Geo-Environmental Exposure a Risk Factor for Multiple Sclerosis? A Population-Based Cross-Sectional Study in South-Western Sardinia
Source: PLoS One. 2016 Sep 26;11(9):e0163313. doi: 10.1371/journal.pone.0163313 (PMC5036813; doi:10.1371/journal.pone.0163313)
Supplement: S2 File — (DOC) [file pone.0163313.s002.doc]

**S1. Study area and geo-data**

***Study area and epidemiological data***

The study was conducted in the South-Western part of Sardinia, which is located between latitudes 39° 26’ and 39° 00’ and longitudes 8° 42’ and 8° 18’, covering an area of 1935.6 km^2^. On 31 December 2007 (prevalence date), the area numbered 138,765 inhabitants (70,627 females and 68,138 males), 3.32% of whom were below 5 years of age and 19.03% over 65 years of age. Within the area there are 25 municipalities, which include the two major cities of Iglesias and Carbonia (Figure 1, main text).

***Geochemical data***

Sardinian analytical data is mainly based on samples collected from previous geochemical surveys conducted between 1974-2006 [1-10].

Through the sampling of several environmental media supports and their chemical analysis, the present research activity has led to the creation of a large database, containing thousands of individual datum, mostly from stream sediments (more than 33k samples), but also from rocks and soils. The stream sediment samples were collected and stored in a geochemical archive at the University of Cagliari. A stream-sediment-type sampling, which is widely used for mineral prospecting, has the advantage of effectively representing the overall geochemical print of a wide portion of territory such as a stream basin.

The samples collected were analyzed by using different analytical procedures, of which the most commonly applied were AAS (Atomic Absorption Spectroscopy), INAA (Instrumental Neutron Activation Analysis), ICP (Inductively Coupled Plasma) and XRF (X-Ray Fluorescence spectroscopy) (Valera P. et al., 2015), It should be noted that the analytical data of the samples taken from the geochemical campaigns have not changed over time, as they still represent typical concentrations of lithologies outcropping in those territories.

Analytical data from stream sediment samples was chosen because it is the most representative among the different sampling media relative to human health [11-12]. In fact, these kinds of samples are ideal to determine the geochemical status of entire alluvial basins [10].

The stream sample dataset has a high variation of spatial density, with an average of approximately 2.2 samples/km^2^. Using a regular grid of approximately 2 × 2.5 km, the entire island territory was divided into rectangles showing the analytical results from stream sediment samples dating to 2006, with at least one sample inside each rectangle.

Finally, the geochemical data reported in this study represent averages of the analytical data in each spatial unit (municipal areas). This data is independent of the location of the samples, i.e., of the other spatial units. In addition, the averages (instead of the median or other position indices) were computed because they are easily influenced by the outliers caused by the ore deposits.

***Geographical data***

Concerning the geographical data, the solar UV exposure data represents the percentage of the municipal areas exposed to the south; it was obtained from the ratio between southern exposure surface and municipal area. This was possible by means of the GIS processing of a DTM (Digital Terrain Model), with an accuracy of 250 meters (i.e., a square of 250 meters on each side). Due to the characteristics and dimensions of the research area, slope and latitude were not considered.

The urbanization data was obtained by extrapolation from two shape files available from the “Regione Autonoma della Sardegna” website (http://www.sardegnageoportale.it). In particular, the “usoSuolo2008CI” and “usoSuolo2008CA” files, which were then converted to .DXF files; the urban spatial dimension was then calculated for each municipality using the AutoCAD software. Finally, the municipal surface percentage of urbanization was obtained from the ratio between the urban and municipal areas.

**References**

1. Marcello A, Pretti S, Salvadori I. I primi risultati delle ricerche sul terreno effettuate nell’ambito del Programma Generale Straordinario avviato dell’EMSa. Notiziario tecnico-economico dell’Ente Minerario Sardo, N° 2, Cagliari. 1974.
2. Marcello A, Pretti S, Salvadori I. Le prospezioni geominerarie in Sardegna: la prospezione geochimica strategica. Boll. Servizio Geologico d’Italia. 1978; 99, 277-310.
3. Marcello A, Pretti S, Salvadori I. Le prospezioni geominerarie in Sardegna. La prospezione geochimica tattica. Notiziario tecnico-economico dell'Ente Minerario Sardo. N° 3-4. Cagliari. 1978.
4. Grillo SM, Mazzella A, Melis F, Porcu R, Pretti S, Rivoldini S, et al. Mineralizzazioni a solfuri associate alle vulcaniti terziarie della Sardegna – Nota I: primi risultati della prospezione dell’area di Perdaxius. Rendiconti Società Italiana di Mineralogia e Petrografia, 1986; 41(2), 369–383.
5. Maccioni L, Marchi M, Padalino G, Pretti S. Preliminary geochemical exploration in semiarid climate: the case of a porphyry – type occurrence in Sardinia (Italy). Journal of Geochemical Exploration. 1992; 42, 261-272.
6. De Vivo B, Boni M, Marcello A, Di Bonito M, Russo A. Baseline geochemical mapping of Sardinia (Italy). J. Geochem. Explor. 1997; 60,77-90.
7. De Vivo B, Boni M, Marcello A, Costabile S, Di Bonito M, Russo A. Cartografia geochimica della Sardegna. In: Cartografia geochimica ambientale. Primi esempi di applicazione: Calabria, Monti Peloritani, Sardegna e Toscana meridionale. De Vivo B, Riccobono F and Sabatini G, editors. Mem. Descr. Carta Geol. d’It. 1998; 55, pp. 97-106.
8. Marcello A, Pretti S, Valera P. The Cagliari, Nuoro, and Sassari sheets of the geochemical map of Italy: explanatory notes. In Ottonello G and Serva L, editors. Geochemical Baselines of Italy. Pacini Editore S.p.A.; 2003; pp. 261-285.
9. De Vivo B, Boni M, Lima A, Marcello A, Pretti S, Costabile S, et al. Cartografia geochimica ambientale e carte d’intervento per l’uso del territorio del Foglio Cagliari, Sardegna meridionale (scala 1:250.000). B. De Vivo Editor. Mem. Descr. Carta Geol. d’It. 2006; 69, 5–38.
10. Marcello A, Muscas F, Pretti S, Valera P. Il ruolo delle campionature "stream sediments" nella definizione dei tenori elementali normali del territorio: esempio del Foglio IGMI 549 - Muravera. In: GEOBASI - Il Foglio IGMI N° 549 – Muravera. Pisa: Pacini Eds, 2008.
11. Valera P, Zavattari P, Albanese S, Cicchella D, Dinelli E, Lima A, et al. A correlation study between multiple sclerosis and type 1 diabetes incidences and geochemical data in Europe. Environ Geochem Health. 2014; 36:79–98.
12. Valera P, Zavattari P, Sanna A, Pretti S, Marcello A, Mannu C, et al. Zinc and other metals deficiencies and risk of type 1 diabetes: an ecological study in the high risk Sardinia island. PLoS ONE. 2015; 10(11): e0141262.
