# Supplementary material for: Is Geo-Environmental Exposure a Risk Factor for Multiple Sclerosis? A Population-Based Cross-Sectional Study in South-Western Sardinia
Source: PLoS One. 2016 Sep 26;11(9):e0163313. doi: 10.1371/journal.pone.0163313 (PMC5036813; doi:10.1371/journal.pone.0163313)
Supplement: S3 File — (DOC) [file pone.0163313.s003.doc]

***SUPPLEMENTAL MATERIAL***

***Heavy metals and geographical factors***

We reported the concentration of heavy metals by SWS municipality (Table 1S). Across the 25 municipalities, Buggerru reports very high average values of lead and Zn, greater than 4000 and 9000 ppm, respectively. This is due to mining activity on ore bodies at upstream sample sites. Furthermore, Gonnesa also points out very high average values of Pb and Zn (>4000 ppm), probably due to the past exploitation of ore bodies in the area. Notably, the municipality of Portoscuso also has record outlier values, greater than 1000 ppm, probably due to Pb-Zn ores present in the Paleozoic rocks and mobilized by leaching. In addition, particularly for Pb and Zn, all the deposits and mineralized manifestations (ore bodies and mining areas in Figures 1 and 2 of the main text) are highlighted from the high standard deviations (Table 1S). Finally, Domusnovas had the highest Cu concentration (64.12 ppm), while Carloforte the smallest value (10.24 ppm) (range~ 50 ppm).

In addition, Table 1S shows solar UV exposure and urbanization percentages. Musei, San Giovanni Suergiu and Tratalias are the municipalities with the highest southern exposure percentages: 96.6%, 81.6% and 80.5%, respectively, due to the morphology of municipal surfaces. Finally, Carbonia (4%) and Portoscuso (3.97%) show high urbanization percentages, while Piscinas has the smallest (0.24%). The urbanization percentage is normally linked to industrial activity, current and/or previous; for example, Carbonia in the case of coal exploitation and Portoscuso with industrial processing activities.

**Table 1S. Concentrations of heavy metals, solar UV exposure and urbanization by municipality**

| **Municipalities (area in km^2^)** | **Cobalt (Co)**  **ppm** | **Chromium (Cr)**  **ppm** | **Copper (Cu)**  **ppm** | **Nickel (Ni)**  **ppm** | **Lead (Pb)**  **ppm** | **Zinc (Zn)**  **ppm** | **Number of**  **sampling sites** |  | **% sun UV exposure** | **% of urbanization** |
| --- | --- | --- | --- | --- | --- | --- | --- | --- | --- | --- |
|  |  |  |  |  |  |  |  |  |  |  |
| **BUGGERRU (48.8)** | 18.87 (17.36) | 68.76 (20.65) | 45.74 (51.08) | 33.29 (13.77) | 4686.19 (8744.32) | 9554.83 (19967.62) | 64 |  | 41.21 | 0.51 |
| **CALASETTA (31.1)** | 2.86 (0.37) | 8.88 (1.89) | 12.12 (7.87) | 7.76 (4.13) | 31.20 (15.35) | 73.38 (50.45) | 5 |  | 21.74 | 2.72 |
| **CARBONIA (145.8)** | 14.15 (4.24) | 74.64 (18.50) | 26.18 (6.02) | 29.52 (7.41) | 181.51 (236.61) | 179.17 (156.00) | 107 |  | 62.93 | 4.02 |
| **CARLOFORTE (51.3)** | 5.18 (8.80) | 6.40 (4.74) | 10.24 (18.26) | 5.40 (3.15) | 69.54 (72.32) | 114.01 (113.40) | 14 |  | 45.17 | 3.30 |
| **DOMUSNOVAS (80.5)** | 18.24 (7.22) | 83.95 (23.35) | 64.12 (180.44) | 35.43 (7.05) | 214.65 (309.34) | 330.75 (188.53) | 63 |  | 70.12 | 1.37 |
| **FLUMINIMAGGIORE (108.4)** | 22.14 (10.31) | 85.94 (20.22) | 49.10 (40.90) | 46.66 (25.93) | 309.11 (514.45) | 667.48 (831.02) | 124 |  | 51.87 | 0.59 |
| **GIBA (30.1)** | 11.88 (4.60) | 37.27 (11.20) | 21.35 (13.43) | 19.15 (7.20) | 66.49 (23.37) | 169.61 (81.39) | 6 |  | 49.38 | 2.70 |
| **GONNESA (47.9)** | 12.55 (5.45) | 58.34 (20.90) | 54.99 (45.95) | 29.04 (11.07) | 4487.42 (9039.34) | 6268.93 (14614.99) | 80 |  | 57.44 | 2.04 |
| **IGLESIAS (207.3)** | 15.70 (5.26) | 73.53 (17.31) | 28.23 (8.26) | 29.45 (7.44) | 539.11 (839.07) | 584.46 (1121.84) | 279 |  | 63.21 | 1.78 |
| **MASAINAS (24.3)** | 41.76 (85.52) | 31.26 (14.44) | 41.39 (59.69) | 25.24 (11.94) | 128.12 (90.77) | 170.96 (86.06) | 8 |  | 67.88 | 1.84 |
| **MUSEI (20.2)** | 14.07 (5.09) | 38.67 (24.04) | 36.71 (10.31) | 25.72 (4.57) | 539.52 (312.75) | 905.55 (715.23) | 5 |  | 96.62 | 1.74 |
| **NARCAO (85.4)** | 20.96 (10.61) | 89.18 (16.76) | 35.66 (18.57) | 38.46 (15.62) | 115.21 (52.37) | 273.58 (198.77) | 71 |  | 77.09 | 0.93 |
| **NUXIS (61.7)** | 17.30 (9.34) | 75.09 (30.45) | 33.77 (17.17) | 42.77 (26.53) | 99.19 (32.43) | 304.74 (188.14) | 74 |  | 33.23 | 0.70 |
| **PERDAXIUS (29.9)** | 14.32 (5.22) | 69.73 (16.80) | 24.91 (5.40) | 28.32 (7.08) | 63.45 (20.48) | 106.95 (25.86) | 22 |  | 68.62 | 1.19 |
| **PISCINAS (16.9)** | 13.76 (4.31) | 45.24 (35.49) | 24.92 (16.56) | 27.53 (8.44) | 65.77 (18.29) | 126.19 (36.38) | 5 |  | 29.52 | 0.24 |
| **PORTOSCUSO (38.0)** | 3.35 (2.46) | 25.12 (29.60) | 49.19 (69.06) | 19.95 (25.09) | 1050.33 (1786.45) | 2013.63 (3386.57) | 8 |  | 75.74 | 3.97 |
| **S. ANNA ARRESI (36.6)** | 14.63 (3.30) | 86.00 (14.73) | 24.81 (6.65) | 30.44 (7.00) | 124.38 (69.38) | 245.00 (83.65) | 16 |  | 64.66 | 1.66 |
| **S. ANTIOCO (88.2)** | 8.05 (7.04) | 12.95 (4.52) | 18.84 (17.38) | 10.78 (4.28) | 51.93 (59.79) | 113.04 (68.85) | 13 |  | 45.45 | 1.92 |
| **S. GIOVANNI SUERGIU (72.3)** | 12.93 (10.13) | 31.63 (29.63) | 24.73 (18.48) | 17.39 (5.80) | 94.64 (74.78) | 223.54 (161.54) | 23 |  | 81.56 | 2.58 |
| **SANTADI (116.3)** | 17.38 (5.87) | 85.71 (29.00) | 41.19 (43.51) | 36.57 (14.48) | 100.36 (82.80) | 293.32 (557.46) | 299 |  | 51.10 | 0.57 |
| **SILIQUA (189.8)** | 25.42 (15.03) | 74.00 (33.77) | 39.92 (37.63) | 38.19 (22.81) | 131.38 (242.05) | 258.67 (200.25) | 300 |  | 45.61 | 0.66 |
| **TEULADA (246.1)** | 20.12 (12.60) | 93.45 (23.78) | 48.32 (25.67) | 43.28 (23.25) | 89.53 (82.56) | 297.91 (265.69) | 476 |  | 57.32 | 0.60 |
| **TRATALIAS (30.9)** | 21.05 (6.59) | 41.64 (17.85) | 21.91 (7.60) | 14.45 (4.17) | 44.02 (14.32) | 121.82 (18.38) | 39 |  | 80.53 | 2.82 |
| **VILLAMASSARGIA (91.3)** | 20.47 (14.34) | 88.75 (44.11) | 35.71 (31.42) | 34.55 (31.10) | 157.41 (462.28) | 218.93 (273.22) | 150 |  | 26.10 | 0.76 |
| **VILLAPERUCCIO (36.5)** | 17.35 (5.00) | 41.13 (15.94) | 32.87 (36.35) | 16.37 (7.27) | 174.20 (476.46) | 327.68 (494.65) | 46 |  | 70.50 | 0.71 |
|  |  |  |  |  |  |  |  |  |  |  |
| **MEAN (SD)*** | 16.18 (7.81) | 57.09 (27.83) | 33.88 (13.41) | 27.43 (11.22) | 544.59 (1236.9) | 957.76 (2182.0) |  |  | 57.38 (18.83) | 1.68 (1.11) |

Geochemical data is expressed as an average (standard deviation) of the sampling sites for each municipal area. Geographical data is expressed as a municipal percentage.

ppm = parts-per-million

* per municipality
